# The *cis*-acting replication element of the Hepatitis C virus genome recruits host factors that influence viral replication and translation

**DOI:** 10.1038/srep25729

**Published:** 2016-05-11

**Authors:** Pablo Ríos-Marco, Cristina Romero-López, Alfredo Berzal-Herranz

**Affiliations:** 1 Instituto de Parasitología y Biomedicina López-Neyra, (IPBLN-CSIC). PTS Granada, Avda. del Conocimiento s/n, Armilla, 18016 Granada, Spain

## Abstract

The *cis*-acting replication element (CRE) of the hepatitis C virus (HCV) RNA genome is a region of conserved sequence and structure at the 3′ end of the open reading frame. It participates in a complex and dynamic RNA-RNA interaction network involving, among others, essential functional domains of the 3′ untranslated region and the internal ribosome entry site located at the 5′ terminus of the viral genome. A proper balance between all these contacts is critical for the control of viral replication and translation, and is likely dependent on host factors. Proteomic analyses identified a collection of proteins from a hepatoma cell line as CRE-interacting candidates. A large fraction of these were RNA-binding proteins sharing highly conserved RNA recognition motifs. The vast majority of these proteins were validated by bioinformatics tools that consider RNA-protein secondary structure. Further characterization of representative proteins indicated that hnRNPA1 and HMGB1 exerted negative effects on viral replication in a subgenomic HCV replication system. Furthermore DDX5 and PARP1 knockdown reduced the HCV IRES activity, suggesting an involvement of these proteins in HCV translation. The identification of all these host factors provides new clues regarding the function of the CRE during viral cycle progression.

Hepatitis C virus (HCV) infection is controlled by a number of host and viral factors (RNA and proteins) that cooperate to initiate the different steps of the viral cycle. The use of secondary and tertiary, structurally conserved, functional genomic RNA elements capable of interacting with proteins and other RNA domains affords important advantages in terms of propagation efficiency. These genomic RNA elements direct and regulate translation, replication and encapsidation. The HCV genome is a single stranded, positive RNA molecule of ~9.6 kb that codes for a single open reading frame (ORF), flanked by untranslated regions (5′ and 3′UTR) of conserved sequence and structure^1^,^2^. These regions play essential roles in the progress of the viral cycle.

HCV protein synthesis initiation is guided by a subset of regulatory structural RNA elements that define an internal ribosome entry site (IRES)^3^,^4^, mostly located at the 5′UTR. The IRES operates via a different mechanism to that used by most cellular mRNAs. HCV protein accumulation determines the initiation of the replication process mediated by the 3′UTR^5^,^6^,^7^. This region contains RNA domains of conserved sequence and structure^6^,^8^ that are specifically recognized by viral RNA-dependent RNA polymerase NS5B^9^ and other host and viral factors^10^,^11^,^12^,^13^. Further, the 3′UTR acts as an enhancer of IRES function in hepatic cell lines^14^,^15^,^16^.

Other structurally and evolutionarily conserved RNA motifs distinct from those in the UTRs have been reported to act as *cis* signals that modulate essential steps during the viral cycle^17^,^18^,^19^. Among these, the HCV *cis*-acting replication element (CRE), located within the 3′ end of the ORF, consists of three stem-loops: 5BSL3.1, 5BSL3.2 and 5BSL3.3 (Fig. 1a). The establishment of long range RNA-RNA interactions between the 5BSL3.2 and 3′SLII within the 3′UTR, or the Alt sequence upstream of the CRE, is critical for the efficient synthesis of the viral RNA negative strand^20^,^21^. Importantly, the existence of an additional, direct, long-range RNA-RNA contact involving the stem-loop 5BSL3.2 and the essential subdomain IIId of the IRES element has been shown to exert an inhibitory effect on HCV IRES function even in the presence of the enhancer 3′UTR^22^. Thus, a dynamic and complex network of RNA-RNA contacts coordinated by the CRE 5BSL3.2 domain is established throughout the HCV genome. The establishment of a specific interaction may depend on the binding of host and viral proteins, which would also help to bring both ends of the HCV genome into close proximity, favoring the acquisition of a circular topology. Numerous factors have been described to simultaneously interact with the 5′ and the 3′UTR of the viral RNA^12^,^13^,^23^, but little is known about the role of CRE in the recruitment of cellular proteins^24^. Given the involvement of the CRE in both viral translation and replication, it seems likely that host factors previously shown to influence these processes also associate with the CRE to promote or stabilize different RNA-RNA contacts. Indeed, the CRE could act as a natural decoy, sequestering or limiting the amounts of certain proteins, thus inhibiting or promoting different steps of the viral cycle and the transitions between them.

The present work provides evidence that the CRE associates with a collection of cellular proteins, most of them reported here for first time, which allows them to connect with the HCV genome. The identification of these proteins by liquid chromatography coupled to mass spectrometry (LC-MS/MS) showed that the CRE is pulled down with a pool of proteins with different biological functions, some of them previously identified as IRES- or 3′UTR-interacting proteins. Many of these proteins bear RNA-recognition motifs (RRMs) within their sequences. Additionally, *in silico* binding predictions suggested that subdomain 5BSL3.2 plays a role in the proteins recruitment. Via knockdown experiments it was confirmed that protein heterogeneous nuclear ribonucleoprotein A1 (hnRNPA1) and High Mobility Group Box 1 (HMGB1) exert biological effects on viral replication whereas DEAD box protein 5 (DDX5) and Poly [ADP ribose] polymerase 1 (PARP1) play a role in HCV translation. Given the involvement of these proteins in different pathways of cell metabolism, the present results support the idea that CRE plays a biological function in the viral cycle by interacting with the host factors here identified. This work provides the first evidence of association of a pool of host cell factors with HCV genome through CRE and the involvement of some of them in different stages of the HCV cycle.

## Results

### Identification of cellular proteins that associate with CRE

**Figure 1:**
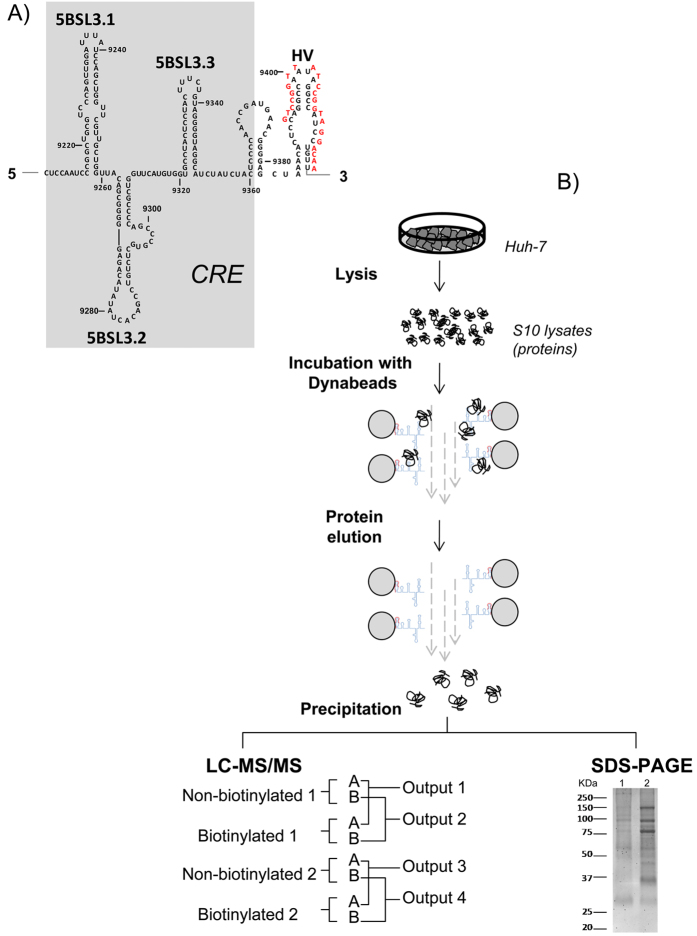
HCV CRE structure and pull-down strategy. (**A**) HCV genome sequence corresponding to the C + HV fragment containing the CRE structure and the downstream region for the hybridization of the oligonucleotide used in the pull-down experiments (red). (**B**) Proteomic method for identifying CRE-interacting candidates. Binding reactions between Huh-7 S10 lysates and CRE were incubated in the presence of streptavidin-coated magnetic beads (Dynabeads) and pulled-down. Some 1/3 of the sample was subjected to SDS-PAGE and the protein stained. Line 1: control samples corresponding to CRE linked to non-biotinylated asHCV-9414; Line 2: positive samples corresponding to CRE linked to biotinylated asHCV-9414 (b-asHCV-9414). Some 2/3 of the samples were digested, analyzed by LC-MS/MS, and compared two by two as explained in Methods.

To identify eukaryotic proteins able to interact with the HCV CRE, pull-down assays were performed involving CRE and Huh-7 cell lysates. To this end, a genomic HCV RNA fragment comprising the CRE plus the downstream hypervariable region (9181–9414) was transcribed (Fig. 1A). This RNA fragment allows the stem loop conformation of the CRE subdomains, and can be annealed to a biotinylated oligonucleotide at its 3′ end, thus allowing fragment capture by affinity chromatography (Fig. 1A). As a control sample the same RNA molecule was annealed to a non-biotinylated oligonucleotide with the same sequence. The non-biotinylated oligonucleotyde control strategy has already been used by other authors for LC-MS/MS analysis of RNA-protein complexes in the HCV related Japanese Encephalitis Virus, confirming the suitability of this control^25^. S10 lysates were obtained as described in Methods and incubated with the RNA fragment in the presence of streptavidin-coupled electromagnetic beads (Dynabeads) that interact specifically with biotin-containing molecules. To avoid recovering proteins with low binding stability, reactions were performed at low ion concentrations and performing washes under stringent conditions. After washing and elution, some 1/3 of the samples were subjected to SDS-PAGE (Fig.1b). As expected, efficient protein enrichment (compared to the control) was seen for the samples with biotinylated oligonucleotides (Fig. 1B).

To identify the pool of proteins, pull-downs were subjected to trypsin digestion and LC-MS/MS. Biological replicates were compared two by two using PEAKS software, and the proteins enriched in at least three of the four replicates selected (see Methods). As shown in Table 1, more than 50 proteins pulled-down with CRE were identified. In most of the proteins, at least 80% of detected peptides were unique; LC-MS/MS therefore provided very good discrimination. Interestingly, some of these proteins, such as XRCC6, hnRNPA1 and DDX5, have already been proposed to interact with other HCV genome regions and/or proteins^12^,^24^,^26^.

### Functional classification of pulled-down proteins

**Figure 2:**
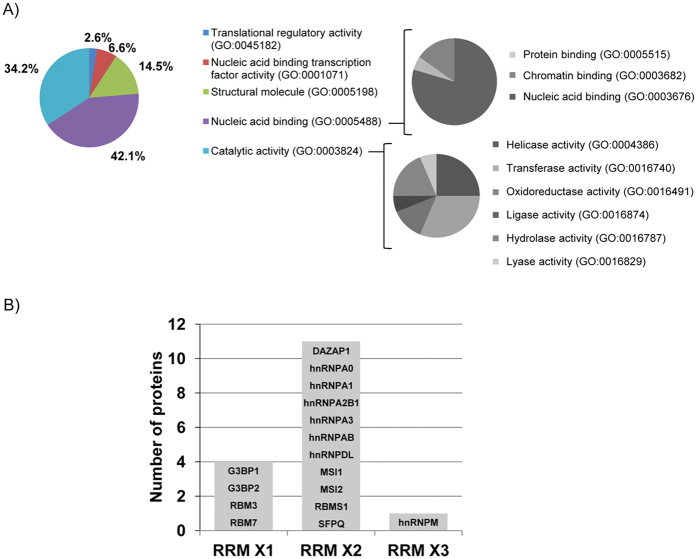
Classification of CRE-interacting protein candidates. (**A**) Panther pie-chart showing the classification of the 55 proteins identified in terms of their molecular function. The two main GO terms were subclassified under the same criterion (pie charts on the right). GO term codes are shown in brackets. (**B**) Classification of RNA-binding proteins via search of the RBPDB database, represented in terms of the number of RRMs. Protein names correspond to the gene names shown in Table 1.

The candidates that interact with CRE were classified using the Panther Classification System gene list analysis tool. Figure 2A shows the CRE-pulled-down protein classification based on “molecular function” criteria. The most represented gene ontology (GO) terms were “binding” (GO:0005488) (42.1%) and “catalytic activity” (34.2%). As expected, the majority of proteins classified as “binding” were “nucleic acid binding” proteins (GO:003676) (79.5% of the binding group); proteins capable of entering a stable union with CRE were therefore enriched. In addition, a large number of proteins with catalytic activities was seen, with transferases and helicases the most strongly represented. Thus, we can hypothesize a possible biological function for either group of proteins in the viral cycle, mediated by their association with the CRE.

An additional classification tool with a broader and both functional and structural focus was used. DAVID bioinformatics resources can extract biological features/meanings associated with large gene lists by simultaneously comparing terms from different annotation categories^27^. The DAVID Clustering Annotation tool revealed eight significant protein clusters for the whole group of pulled-down host factors (Table 2). The cluster with the highest enrichment score (15.9) was composed of terms related to RNA binding motifs and domains; all had very high significance values. Among the proteins clustered as RNA binding proteins were several heterogeneous nuclear ribonucleoproteins (hnRNPs), Ras-GTPase activating-binding proteins (G3BPs) and splicing factors.

**Figure 3:**
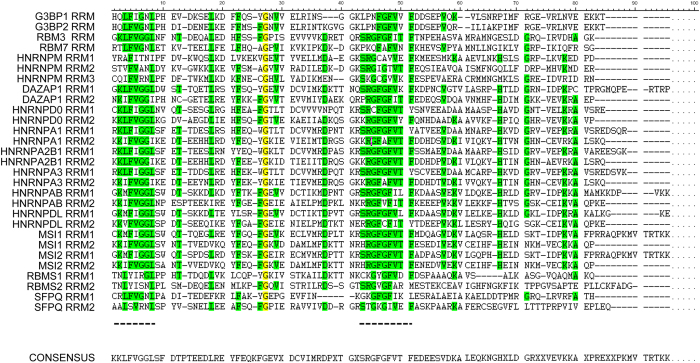
Alignment of RRMs corresponding to the RNA-binding proteins. All RRM sequences were obtained from the Uniprot database and proteins compared using DNAsis software. Complete homology between aminoacids is highlighted in yellow; strong homology is highlighted in green. There are two conserved regions within the first 60 aminoacids of the RRMs. The consensus sequence is represented below.

All the proteins belonging to the RNA-binding cluster were checked against the RNA-Binding Protein (RBPDB) database (http://rbpdb.ccbr.utoronto.ca/) to identify the type and number of RNA binding domains involved in the protein-CRE interaction. Interestingly, all showed the presence of RRMs in their sequence (Fig. 2B). The presence of just one RRM seems sufficient to allow pulldown with CRE, as seen for G3BP1 G3BP2, RBM3 and RBM7. Several hnRNPs showed two RRMs in their sequence, but only the hnRNPM protein had more than two RRMs. To search for similarities between RRMs, all 29 protein sequences corresponding to these domains were compared, and two highly conserved regions found (Fig. 3). Together, these data suggest that there is a large group of the pulled-down proteins (29 out of the 55) that contain conserved RRMs.

### *In silico* interaction of RNA- and DNA-binding proteins with CRE

**Figure 4:**
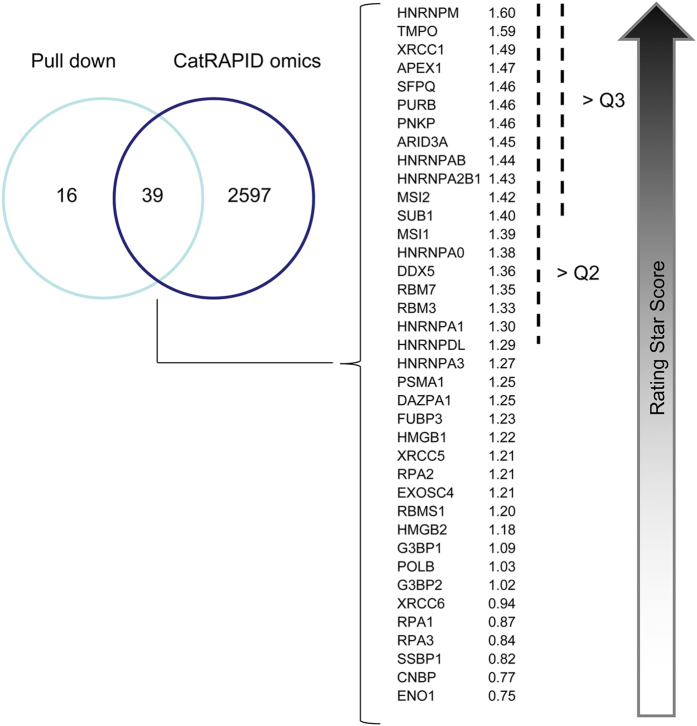
Match between experimental pulled-down proteins and *in silico* associations. Vein diagram showing the number of proteins identified in pull-down experiments and the catRAPID omics output. Of the 55 pulled-down proteins, 39 were identified *in silico* to bind to the CRE sequence. These 39 proteins were ordered by their catRAPID omics Rating Star Score. Scores corresponding to quartile 2 (Q2) and quartile 2 (Q3) from the total of the *in silico* predicted proteins were used as a reference for the 39 coincident host factors.

To further study the associations detected by LC-MS/MS, an *in silico* interaction study was performed, introducing the CRE sequence to the human RNA- and DNA-binding proteome. catRAPID tools estimate the binding propensity of a protein-RNA pair using an algorithm that considers protein and RNA secondary structure, hydrogen bonding, and contribution of van der Waals forces^28^. The CRE nucleotide sequence was compared against the *Homo sapiens* proteome using the catRAPID omics tool, searching for interacting RNA- and DNA-binding proteins. Interestingly, 71% (39/55) of the proteins identified in the pull-down experiments were predicted to bind as well to the CRE sequence (Fig. 4). In addition, among these 39 proteins, 30% fit within the upper quartile score, indicating a good reliability on the identification. The *in silico* binding prediction is in agreement with the pull-down results and reinforces these proteins as CRE-associated factors.

**Figure 5:**
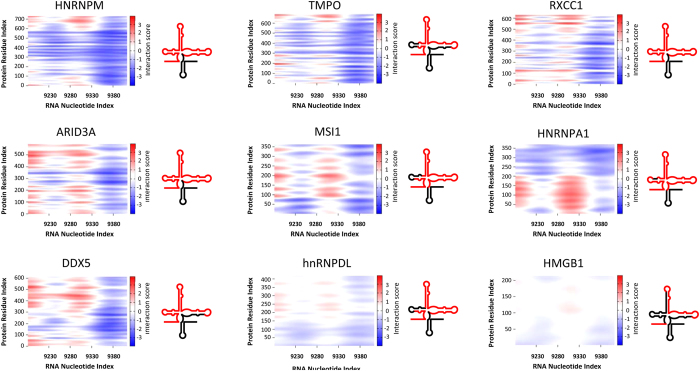
Heat-maps of aminoacid-nucleotide interactions. Nine proteins were subjected to interaction studies against the CRE sequence using the catRAPID graphics tool. Aminoacid-nucleotide interactions for the whole protein and RNA sequences are represented as heat-maps that show interacting score values between −3 and +3. The strongest interacting regions correspond to the red areas; blue areas represent pairs showing weak aminoacid-nucleotide interaction. The CRE regions corresponding to nucleotides with high interaction values are highlighted in red in the chart on the right.

Deeper mapping of the binding sites of the CRE-interacting proteins was performed using nine representative factors detected in both *in silico* and the pull-down experiments. The selected proteins were analyzed using the catRAPID graphics tool, comparing each candidate protein’s whole sequence against the C + HV sequence transcript. Figure 5 shows the aminoacid-nucleotide interaction heat-map for each protein against the CRE. Remarkably, all the proteins examined showed very similar CRE interaction patterns. The majority of the proteins most strongly interacted with the region comprising nucleotides 9280–9330. This region includes the 5BSL3.2 apical loop and the 5′ end of 5BSL3.3. In contrast, the nucleotides downstream of the CRE have no apparent role in these interactions; their catRAPID scores were low. This indicates that, in the C + HV fragment, nucleotides mainly belonging to the CRE show protein binding functionality. It should be noted that, for the RNA-binding proteins, the interacting aminoacids clearly belonged to their RRMs. For instance, hnRNPA1 (RRMs at 14–97 and 105–184) showed its strongest interaction within the first 200 aminoacids. In addition, hnRNPM showed strong overlapping between its RRMs and the interacting regions, especially at its third RRM (653–729). This was the case too for the second RRM of MSI1 (109–186) and both the RRMs of the hnRNPDL (148–230 and 233–312). Together, these findings suggest a clear involvement of CRE 5BSL3.2 and 5BSL3.3 in CRE-protein association, mediated by RRMs in the case of the RNA-binding proteins.

### Effect of gene modulation on HCV replication

**Figure 6:**
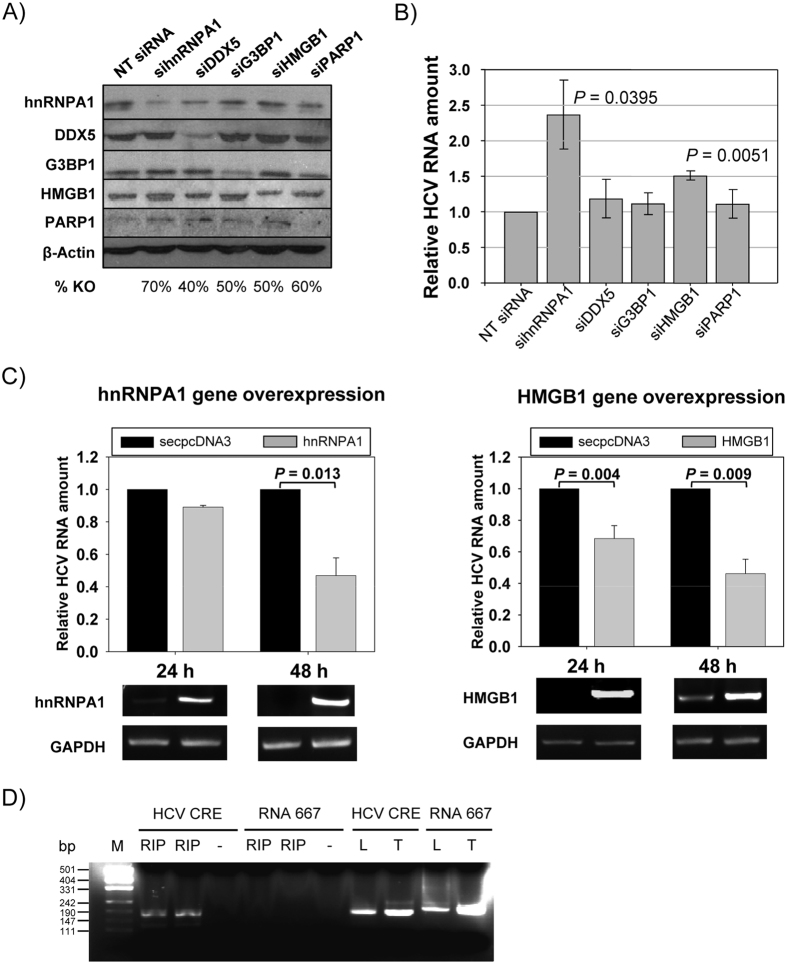
Effect of mRNA knockdown in HCV replication. Replicon-containing cells (Huh-7-NS3-3′) were transfected with siRNAs directed against either hnRNPA1, DDX5, G3BP1, HMGB1 or PARP1, or with a non-targeting siRNA. After 48 h the cells were collected and RNA or protein extracted as described in Methods. (**A**) Protein lysates from Huh-7-NS3-3′cells transfected with siRNA were resolved by SDS-PAGE and immunoblotted with specific antibodies for the assayed proteins or for a β-actin control. (**B**) RNA lysates were retrotranscribed and cDNA corresponding to HCV replicon amplified by qPCR. Changes in replicon levels were represented as fold changes normalized to GAPDH. Standard deviation is displayed for each siRNA. Significant differences between siRNAs and non-targeting siRNA are indicated above the corresponding bar (p < 0.05 in a two-tailed Student’s t-test). (**C**) Overexpression of hnRNPA1 and HMGB1 coding genes. Replicon levels, normalized to GAPDH, are represented after 24 h and 48 h after transfection. *P* values indicating the statistical significance are included. Values are the mean of three independent experiments. A picture of a representative agarose gel showing the relative hnRNPA1 and HMGB1 mRNA levels to the GAPDH mRNA ones analyzed by RT-PCR is shown under each bar graph. (**D**) Immunoprecipitation of CRE and RNA 667 with anti-hnRNPA1 antibody. After RIP, samples were subjected to RT-PCR and resolved in a 2% agarose gel. Immunoprecipitated (RIP) samples from cells transfected with CRE and RNA 667 were run in duplicates with a negative sample without template (−). As positive controls, not immunoprecipitated lysates (L) and direct RNA template (T) were retrotranscribed and amplified. pUC19 plasmid digested with *Msp*I was used as DNA size marker (M). Fragment sizes, in bp, are indicated on the left.

To study the potential effect of these proteins in the viral replication, gene silencing assays were performed. Six proteins were selected based on previously suggested HCV interactions or a possible role in the viral cycle, and knockdown experiments performed on them. To this end, a cell line carrying a stable HCV subgenomic replicon (Huh-7 NS3-3′) was transfected with siRNAs designed against the mRNA of these proteins. For this purpose those siRNA sequences that have already been proved to be effective were used^29^,^30^,^31^. The specific effect of each siRNA was confirmed by immunoblotting (Fig. 6A). As can be seen, all the siRNAs reduced their target protein levels (from 40% to 70%) without significantly affecting any of the other assayed proteins. RNA was extracted from cells treated with the different siRNAs and HCV replicon levels quantified by qRT-PCR (Fig. 6B). Interestingly, hnRNPA1 gene knockdown resulted in a two-fold increase in HCV replicon levels compared to those associated with a non-targeting siRNA. The same tendency, but with only a 1.5-fold increase, was observed in cells transfected with siHMGB1. No significant effect was detected for G3BP1, DDX5 and PARP1 knockdown compared to results obtained with non-targeting (NT) siRNA.

To confirm the knockdown results we selected hnRNPA1 and HMGB1 and cloned their respective coding genes into the pcDNA3 eukaryotic expression vector. Huh-7 cells were transfected with pcDNA3 (as a control) or the corresponding hnRNPA1- or HMGB1-pcDNA3 constructs. Interestingly, 48 h after transfection a significant inhibition of the replicon levels was detected in both cases (Fig. 6C), reinforcing the results obtained in the knockdown experiments for these two proteins. These results suggest that hnRNPA1 and HMGB1 negatively regulate HCV replication.

Moreover, direct interaction of hnRNPA1 with CRE was assayed through an RNA-immunoprecipitation protocol (RIP). Results shown in Fig. 6D shows that CRE RNA fragment, but not a control RNA 667, was amplified in immunoprecipitated samples after RIP using an anti-hnRNPA1 antibody. This experiment confirms a direct interaction between hnRNPA1 and HCV CRE.

### DDX5 and PARP1 knockdown reduce viral translation efficiency

**Figure 7:**
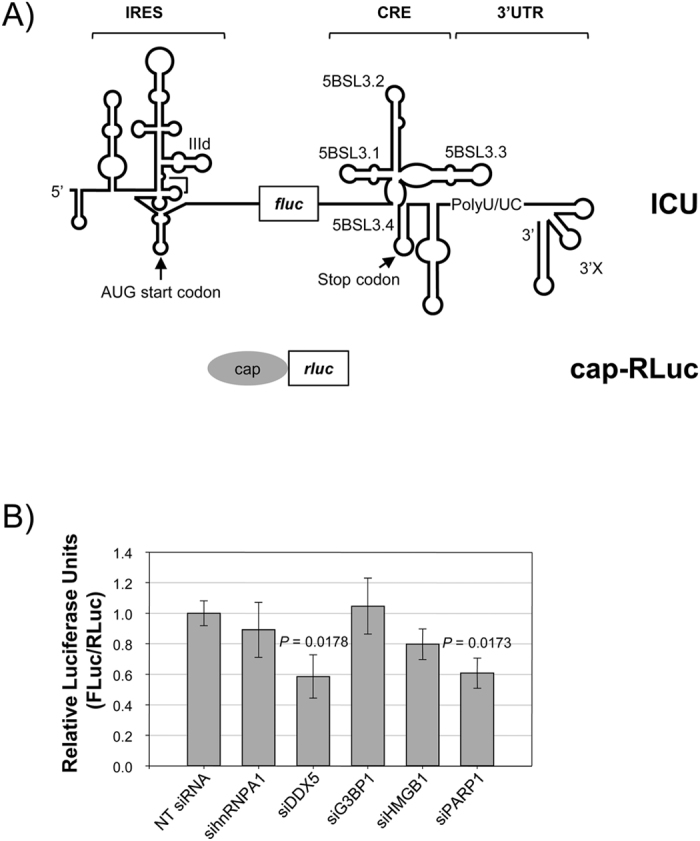
HCV IRES activity in Huh-7 cells treated with siRNAs. (**A**) Scheme of the ICU and Cap-RLuc RNA constructs. ICU is composed by HCV IRES, CRE and 3′UTR regions flanking the reporter gene of the *Firefly* luciferase. cap-RLuc contains *Renilla* luciferase RNA, capped during *in vitro* transcription. (**B**) Huh-7 cells were treated with the indicated siRNAs and transfected with a mix containing 1 μg FLuc-ICU and 0.25 μg cap-RLuc per well. Luciferase activity was measured after 4 h and translation efficiency calculated as FLuc/RLuc activities. Standard deviation is displayed for each siRNA. Significant differences between siRNAs and non-targeting siRNA are indicated above the corresponding bar (p < 0.05 in a two-tailed Student’s t-test).

Since CRE was been shown to long-range interact with the IRES and inhibit its translation function^22^, an RNA construct containing a luciferase reporter gene was generated (ICU), to perform translation assays in knock down cells. This molecule contains the HCV IRES linked to the CRE and 3′UTR through the *Firefly* luciferase gene 22 (Fig. 7A). After protein knockdown with the specific siRNAs, Huh-7 cells were transfected with the RNA construct, (Fig. 7A), and luciferase activity measured. The luciferase sequence of ICU includes its own stop codon, ensuring the correct translation of the reporter protein. A non-related transcript with a cap structure, containing *Renilla* luciferase reporter (cap-RLuc) was used for normalization of translation data. Translation efficiency at 4 h post-transfection is shown in Fig. 7B. Knockdown of DDX5 and PARP1 showed nearly of 40% of inhibition of HCV IRES activity. This effect was present, but patently lower, in siHMGB1 treated cells. All these data indicate that DDX5 and PARP1 are required for full HCV IRES translation.

## Discussion

Many processes are run by viral genomes through the use of structurally conserved functional RNA domains which form a dynamic, complex network of direct RNA-RNA interactions that may be regulated or stabilized by the recruitment of host or viral factors. In the case of HCV, the CRE region plays an active role in the organization of an RNA-RNA contact network involved in the regulation of viral translation and replication^20^,^21^,^22^,^32^,^33^.

To our knowledge, very few attempts to study CRE-protein association have been made. The present work identifies a pool of 55 host proteins that are pulled-down by an RNA transcript corresponding to the HCV CRE. The majority of these proteins are proposed as HCV-interacting proteins for the first time. Using MALDI-MS, Oakland *et al.*^24^ identified six CRE-interacting proteins in 293T cells. Interestingly, two of these proteins were detected in the present study as well (hnRNPM and XRCC5), and another two (DDX17 and DDX3) are closely related to the reported RNA-helicase DDX5. In the present work, the resolving power of the LC-MS/MS system used not only allowed the identification of many more new proteins, it detected, with high coverage, a large number of unique peptides (Table 1). Moreover, label-free quantification using PEAKS software allowed the positive samples to be compared with the controls and discard any factors that were recovered in a non-specific manner, increasing the reliability of the results. The use of a non-biotinylated oligonucleotide as a control has been successfully used previously^25^ and it constitutes from our belief a good approach to discard unspecific targets.

As would be expected for an RNA-protein pull-down method, most of the proteins identified in this work were classified as nucleic acid binding proteins. This suggests direct association between them and the CRE. However, protein-mediated interactions cannot be ruled out since 6% of the factors were initially termed as protein-binding proteins. Using bioinformatics resources such as DAVID searches, which do not rely exclusively on GO terms, protein pool was clustered into eight different groups; that with highest score corresponded to RNA-binding proteins (Table 2). Interestingly, all this cluster′s proteins shared the presence of one or more RRM, also termed RNA binding domains, within their sequences. The comparison of all RRMs showed two clear, highly conversed regions that matched consensus sequences previously defined as ribonucleoprotein domain RNP1 and RNP2 (see consensus sequence in Fig. 3)^34^. The high degree of conservation in these sequences suggests they are involved in the association with the CRE. One example of this is the La protein, that interact with HCV IRES through its RRM and promote linkage between the 5′ and 3′ ends of the viral genome^35^.

The catRAPID algorithm, which studies RNA-protein interactions based on their sequences and secondary structure, strengthened the reliability of identification of the pulled-down proteins, even though some are termed DNA-binding proteins. HMGB1 provides an example of these proteins reported to bind with high affinity to branched RNA structures^36^. Another interesting feature of a group of proteins is their ability to bind AU-rich elements (AREs), The present work identified several proteins reported by other authors to act as ARE binding proteins (ARE-BPs): hnRNPA1^37^ DAZAP1^38^, hnRNPA0^39^, hnRNPA3^39^,^40^ and RBM3^40^. It is noteworthy that CRE has exposed AU-rich elements, i.e., the ARE consensus sequence (-AUUUA-) located in the apical loop of 5BSL3.1, and a non-canonical sequence in the apical loop of 5BSL3.2 (5′-AUAUAU- 3′) (Fig. 1A). This would explain the abundance of recovered peptides corresponding to these proteins after LC-MS/MS.

Considering the involvement of CRE in HCV translation is interesting to remark that two proteins related to the regulation of translation were identified: RNA-helicase DDX5 and eukaryotic translation initiation factor I subunit I (eIF3I). In fact, other subunits such as eIF3E, eIF3F and eIF3H were also detected in one or two experimental replicates, and other proteins related to translation (such as eIF1A, eIF2A and the 40S/60S ribosomal proteins) were detected in previous pull-down experiments performed under less stringent conditions (data not shown). This is in good agreement with the HCV IRES translation regulation exerted by CRE^22^. The translation efficiency assays performed in this work show a clear involvement of DDX5 in HCV translation. DDX5 knockdown up to a 40% was able to cause a specific decrease of IRES activity of 40% (Fig. 6B). However, this effect was not caused by other siRNAs assays that displayed even higher knockdown effects (e.g. sihnRNPA1 or siG3BP1). Remarkably, other proteins from the DDX5 family (DEAD box) have been shown to promote translation through their helicase activity^41^,^42^. In addition, siPARP1 decreases translation efficiency; however an indirect effect of PARylation inhibition cannot be ruled out because of the high amount of PARP1 substrates that take part in a variety of cell processes^43^. The detection of translation-related proteins in the pulled-down assays plus the described modulation of translation activity of HCV CRE-containing RNA constructs, suggests an association between the translational machinery and the CRE when the cellular scenario is suitable. Further, a group of nine proteins (with hnRNPs, RNA-binding Musashi homologues, RNA-binding proteins and DAZ-associated protein among them) have been classified as structural constituents of ribosomes (GO:0003735), and to belong to the structural molecule activity group (GO:0005198). This reinforces that CRE is able to interact with ribosome components and modulate viral translation through association with host translation factors. Experiments designed to see whether this is the case are currently underway.

Finally, some proteins were selected to check whether they had any important involvement in HCV replication. In this work, replication experiments were performed on a hepatoma cell line containing a subgenomic HCV replicon defective for the viral protease (NS2/NS3). As an advantage, these assays reproduce quite accurately the HCV genome replication, being a good biological model. On the other hand one cannot exclude additional interactions of the assayed proteins with other regions of the HCV genome contained in the replicon, more than the CRE element. In fact some of the proteins tested for their implication in replication have been suggested to interact with other regions of the genome of the HCV (see references in table 1). Regarding the studied candidates, HMGB1 is an antiviral factor that changes its cell location and is secreted to the extracellular medium after HCV infection^44^. In agreement with a previous report showing that blocking of HMGB1 with specific antibodies increases HCV infectivity^44^, the results derived from the knockdown and overexpression experiments suggest that HMGB1 negatively modulates HCV replication in the replicon system. On the other hand, in the present work no significant effect of G3BP1 on HCV replication was detected, although other authors report enhancing effects of G3BP1 on this process^12^,^45^,^46^. These discrepancies, however, could be due to differences in the replicon model used or the amount or strategy of interference RNA employed. G3BP1 has been described to bind HCV RNA polymerase NS5B^47^ suggesting that it possesses both RNA- and protein-interacting sites, and that it may be able to regulate replication. It should be noted that G3BP1 has been reported to associate with viral genome 3′ elements that regulate replication in Dengue virus, a member of the family *Flaviviridae*^45^. All this together suggests G3BP1 to be a cellular host factor that can be hijacked by the genome of RNA viruses, with a consequent effect on viral replication. Finally, a protein with high antireplicative activity was found: hnRNPA1. Here we report the direct association of hnRNPA1 to the HCV CRE by the pulldown and the immunoprecipitation experiments. This ribonucleoprotein has previously been reported to bind as well to the viral polymerase NS5B^48^. In the latter work, the binding of hnRNPA1 to the 5′ and 3′UTR (downstream of CRE) was also shown. Since CRE plays a role in both NS5B binding^49^ and in viral 5′-3′contacts^21^,^22^, one might hypothesize that the binding of hnRNPA1 to CRE would have an effect on HCV replication. In the present work, hnRNPA1 clearly downregulates HCV replication, one explanation could be that this protein competes with NS5B for CRE binding, thus reducing the efficiency of the viral polymerase. However, due to the limitation of the replicon system, we cannot exclude the possibility of a partial effect of hnRNPA1 knockdown on HCV replication by direct interaction with NS5B or even synergic effects of both actions. Additionally, hnRNPA1 has been reported to act as a splicing factor in the maturation of IFR3, which is involved in the interferon response^50^. The role of hnRNPA1 in HCV resistance to IFN response would certainly be interesting to explore.

In conclusion, the current work reports a number of new HCV RNA-host protein associations. Further work on these factors will be needed to better comprehend their function in the viral cycle.

## Methods

### DNA templates and RNA synthesis

DNA coding for the CRE transcript was obtained by PCR amplification, ensuring the presence of the precise 3′ end. Briefly, T7pC + HV was amplified from plasmid pU3′HCV9181 using the primers T7pHCV-9181^33^ and asHCV9414 (5′-AACAGGATGGCCTATTGGCCTG-3′) to obtain the template for CRE plus the downstream hypervariable region (9181–9414). A shorter amplicon, CRE (9181–9384), was amplified using T7pHCV-9181 and as HCV9384 (AGCTCCCCGTTCATCGGTT) primers. The template for RNA 667 was derived from the pcDNA3 vector (Invitrogen) linearized with *Dra*III^51^. RNA synthesis was performed using the TranscriptAid T7 High Yield Kit (Thermo Fisher Scientific), following the manufacturer’s instructions. The resulting transcript was purified as previously described^52^. The RNA concentration was determined by UV spectrophotometry (A_260_) and the degree of protein and carbohydrate/phenolic contamination assessed from the A_260_/A_280_ and A_260_/A_230_ ratios respectively. The integrity of the RNA was confirmed by denaturing agarose-formaldehyde gel electrophoresis.

RNA construct (ICU) containing a luciferase reporter gene flanked by the HCV genomic ends was obtained by amplification of pGLICU plasmid with 5′pT7HCV and 3′HCV primers^22^, *in vitro* transcription and purification as explained above. The generation of a capped RLuc RNA was generated as previously described^51^.

### Cell culture

The cell lines used in this study were a gift from Dr. R. Aldabe (University of Navarra, Spain). The human hepatoma Huh-7 cells were maintained in Dulbecco’s modified Eagle medium (DMEM) supplemented with 10% heat-inactivated fetal bovine serum and 1 mM sodium pyruvate, at 37 °C in a 5% CO_2_ atmosphere. The knockdown assays were performed in the named cells and in a human hepatocarcinoma cell line harboring an HCV subgenomic replicon system (Huh-7 NS3-3′)^53^,^54^. Replicon cells were maintained in DMEM supplemented with 20% heat-inactivated fetal bovine serum and 0.5 mg/ml G-418 under the same conditions as described for the Huh-7 cells.

### Cell lysate preparation

S10 fractions of Huh-7 cell lysates for use in pull-down assays were essentially prepared as previously described^55^, with some modifications. Briefly, around 5 × 10^7^ Huh-7 cells were grown as described above to reach 100% confluent monolayers. They were then treated with trypsin, washed with 10 volumes of isotonic buffer (35 mM HEPES/KOH, pH 7.6, 150 mM NaCl and 11 mM glucose) and pelleted by centrifugation at 1,000 × *g* for 5 min. Cellular lysis was performed by adding 1.5 volumes of cold hypotonic buffer (20 mM HEPES/KOH, pH 7.6, 10 mM KCl, 1.5 mM MgAc, 1 mM DTT and protease inhibitors) and incubation at 4 °C for 20 min. Cell lysate was homogenized in a glass Dounce homogenizer (25 strokes) and equilibrated with 0.2 volumes of S10 buffer (100 mM HEPES/KOH pH 7.6, 0.6 M KAc, 20 mM MgAc, 25 mM DTT and protease inhibitors). Cellular debris was removed by centrifugation at 10,000 × *g* for 10 min. The resulting cleared lysates contained around 30 A_280_ units ml^−1^.

### Isolation and identification of CRE-interacting proteins

Transcript C + HV (500 pmol) was annealed to 1 nmol of the 5′ biotinylated oligonucleotide (b-asHCV-9414) (Eurofins MWG Operon) or to the respective unlabeled asHCV-9414 for control reactions (sequence detailed in DNA template and RNA synthesis). It was then incubated with Huh-7 S10 cell extracts (~10 mg of proteins) pretreated with 50 μg of tRNA and CL buffer (100 mM HEPES pH 7.6, 50 mM KAc, 1 mM MgAc and 1 mM DTT). Reactions were performed at 25 °C for 20 min to allow for CRE-protein interactions to take place. For the affinity chromatography step, 5 mg of streptavidin-coupled Dynabeads^®^ (Invitrogen) were washed and equilibrated following the manufacturer’s instructions. Ribonucleoprotein complexes were captured by incubation of the Dynabeads in the binding reaction in a rotation wheel for 30 min at room temperature. Unbound factors were removed after washing three times with buffer B (0.1 M NaCl and 0.1% Tween^®^ 20) pre-warmed at 37 °C. CRE-protein complexes were eluted from the beads by heating for 2 min at 95 °C in the presence of 95% formamide and 10 mM EDTA. The recovered polypeptides were precipitated in methanol/chloroform. Some 2/3 of the sample volume was aliquoted for LC-MS/MS analysis and the rest used in SDS-PAGE. For the latter, samples were resuspended in Laemmli buffer 1X (10 mM Tris-HCl pH 6.8, 5% SDS, 2 mM EDTA, 5% Glycerol, 1% β-mercaptoethanol, 0.1 mg/ml Bromophenol blue) and loaded onto a polyacrylamide gel (5% stacking, 12% resolving). Proteins were stained by Sypro-Ruby (BioRad) and scanned in a Typhoon 9400 scanner (GE Healthcare). Both biotinylated and non-biotinylated reactions were conducted in duplicate (biological samples = 4).

### Mass spectrometry analysis

The rest of the samples were resuspended, digested and run in LC-MS/MS following a protocol similar to that used by others^56^,^57^ with minor modifications^58^. The specifics of this protocol are depicted in Supplementary Note S1. Each replicate was run twice, independently (two technical replicates per sample). Protein enrichment was established by PEAKS 6 software as protein ratio ≥1.00:0.50 in biotinylated samples versus the controls. Only proteins with >15% coverage in positive samples (biotinylated) were finally selected. The number of recovered peptides, coverage and ratios of positive samples were calculated as average of the four corresponding technical replicates. The list of the proteins enriched for each comparison is shown in Supplementary Table 1.

### Bioinformatics resources

The Panther gene list analysis tool was used to classify the pulled-down proteins^59^. The list for analysis included the Uniprot IDs shown in Table 1 (or analogue IDs in the very few necessary cases). Proteins were classified in gene ontology (GO) terms related to “molecular function”, and their proportions represented in a pie chart. Successive classification was performed for the two most represented groups.

The same protein list was examined using the Database for Annotation, Visualization and Integrated Discovery (DAVID) v6.7 bioinformatics resources (http://david.abcc.ncifcrf.gov/home.jsp). Functional annotation clustering of the protein list was performed under the highest classification stringency. A total of 13 annotation categories were selected (by default) for the search. As indicated by Huang and co-workers^27^, only clusters with an enrichment score of ≥1.3 and a p value of <0.05 were taken into account for further analysis.

The catRAPID webserver was used for predicting RNA-protein interaction (http://service.tartaglialab.com/page/catrapid_group). The CRE sequence was run against the *Homo sapiens* nucleotide-binding proteome using the catRAPID omics tool. A search was performed for RNA- or DNA-binding full-length proteins. Proteins were ordered based on their Rating Star Score, which considers the protein-RNA Interaction Propensity score (i.e. the interaction probability) and the presence of RNA/DNA binding domains and motifs. Additionally, nine different FASTA protein sequences, taken from the Uniprot database, were run (using the catRAPID graphic tool) against the complete CRE sequence, and nucleotide-aminoacid interaction heat-maps obtained.

### Protein knockdown mediated by RNA interference and subgenomic HCV RNA quantification

Huh-7 NS3-3′ cells were cultured (300,000 cells/well) in 6 well-plates to obtain a confluence of at least 80% after 24 h. Cells harbouring a subgenomic HCV replicon were transfected with small interference RNAs (siRNAs) designed against the mRNAs of the proteins of interest. For hnRNPA1, DDX5, G3BP1 and HMGB1, a pool of four targeting siRNAs for each protein was designed (Dharmacon). For PARP1 inhibition, a specific siRNA (Sigma) was used. As a negative control, a single non-targeting (NT) siRNA (Dharmacon) was selected. siRNAs were mixed with Transfectin^TM^ solution in Opti-MEM^®^ medium. The transfection mixture was added to the Huh-7 NS3-3′ cells in an antibiotic-deprived medium to achieve a final siRNA concentration of 50 nM. After 48 h, the cells were collected for RNA or protein extraction.

RNA extraction was performed using Trizol reagent^54^. Primer extension was achieved using the High Capacity cDNA Reverse Transcription Kit (Thermo Fisher Scientific). Briefly, RNA extracted (40 ng) was annealed to random primers in a 10 μl volume reaction, at 95 °C for 2 min, and at 16 °C for 15 min. cDNA was retrotranscribed in a total volumen of 20 μl at 37 °C for 30 min following manufacturer instructions. Enzyme inactivation was performed at 85 °C for 5 min. Intracellular HCV replicon RNA levels were measured as previously described and normalized with those obtained for the internal mRNA encoding GAPDH^54^. The HCV fragment was amplified by PCR over 40 cycles (5 s at 95 °C and 5 s at 58 °C) in a CFX96 Thermal Cycler (BioRad).

For protein extraction, cells were scraped after adding 500 μl of cold PBS, and centrifuged at 2500 rpm. The resulting pellet was resuspended in 200 μl of protein Lysis Buffer (50 mM Tris-HCl pH 7.4, 150 mM NaCl, 1% Triton X-100, supplemented with protease inhibitor cocktail tablets) for 30 min at 4 °C. Lysates were centrifuged at 12,500 rpm and the supernatant stored at −20 °C. The protein concentration was measured using BioRad Protein Assay reagent. Proteins were separated by SDS-PAGE and transferred to nitrocellulose membranes in a SemiDry Trans Blot apparatus (BioRad). Membranes were blocked in 5% dry milk in TBS-Tween and incubated with specific primary antibodies against hnRNPA1, DDX5, HMGB1 (Cell Signaling Technology) G3BP1 (Santa Cruz Technology) or PARP1 (a gift from Dr. Oliver, Institute of Parasitology and Biomedicine López-Neyra, Granada, Spain) in 1% BSA-TBS/Tween solution at 4 °C overnight. Secondary HRP-linked antibodies were added for 1 h. Chemiluminescence was then detected using the Clarity^TM^ Western ECL Substrate Kit (BioRad).

### Overexpression of hnRNPA1 and HMGB1 coding genes

Total RNA from Huh-7 cells was extracted using Trizol reagent. Primer extension was performed as described above using instead an oligodT and annealing at 25 °C. cDNA was amplified by PCR using 5′HMGB1-EcoRI (GCGTATGAATTCATGGGCAAAGGAGATCCTAAGAA) and asHMGB1 (GCGTATCTCGAGTTCATCATCATCATCTTCTTCT) primers for HMGB1 and 5′A1 (GCGTATGGATCCATGTCTAAGTCAGAGTCTCCTAAA) and asA1 (GCGTATCTCGAGTTAAAATCTTCTGCCACTGCCATA) for hnRNPA1. PCR amplicons were digested with *Eco*RI and *Xho*I for HMGB1 sequence and *Bam*HI and *Xho*I for hnRNPA1 and inserted in pcDNA3 vector digested with the corresponding enzymes. Plasmid isolation was perfomed using Qiagen^®^ Plasmid Maxi Kit (Qiagen). Huh-7 NS3-3′ cells were cultured in 6 well plates and allowed to grow up to 80% confluence, then cells were transfected with 2 μg/well of pcDNA3 (as a control) or pcDNA3-hnRNPA1 or pcDNA3-HMGB1, and total RNA was extracted 24 h or 48 h after transfection using Trizol reagent. Quantification of HCV RNA levels was performed by RT-qPCR as described above. Relative amounts of hnRNPA1 and HMGB1 mRNAs to GAPDH mRNA in the total RNA mix was analyzed by RT-PCR amplification with the following primers: 5′A1-2D (GCGTATGGATCCTCCAGCCAAAGAGGTCGAAGT) and asA1 for hnRNPA1; asHMGB1-A (GCGTATCTCGAGGAACTTCTTTTTTGTCTCCCCTT) and 5′HMGB1 for HMGB1. Amplification products were resolved in a 2% agarose gel.

### RNA-immunoprecipitation (RIP)

The RIP assay was carried out following the protocol described by Niranjanakumari and coworkers^60^. Briefly, Huh-7 cells at 80–90% confluence were transfected with same amount (85 pmol) of CRE (9181–9384) or RNA 667. After 4 h cells were trypsinized, collected in PBS and crosslinked with 5% formaldehyde for 10 min. Quenching with 250 mM Glycine for 5 min was done and cells were washed, pelleted by centrifugation and frozen down at −80 °C. Cells were resuspended (an aliquot stored at −20 °C) in RIPA buffer (50 mM TRIS-HCl, 1% NP-40, 0.5% Sodium deoxycholate, 0.05% SDS, 1 mM EDTA, 150 mM NaCl, and protease inhibitors), sonicated and precleared with Protein A Dynabeads (Cell Signaling Technology) in the presence of 25 μg of tRNA per reaction. Precleared lysates were recovered and exposed to new Protein A Dynabeads previously linked to the anti-hNRNPA1 antibody (which is compatible with immunoprecipitation). Binding was performed for 90 min in rotation and beads were extensively washed (6 times) with high-stringency RIPA buffer (50 mM TRIS-HCl, 1% NP-40, 1% Sodium deoxycholate, 0.1% SDS, 1 mM EDTA, 1 M NaCl, 2 M Urea and 0.2% PMSF). Crosslink was reverted by beads resuspension in 50 mM Tris-HCl, 5 mM EDTA, 10 mM DTT and 1% SDS and heating at 70 °C for 45 min, conducting in parallel an aliquot of the lysates. RNA was extracted and retrotranscribed using the specific primers asHCV9359 (TAGATAGATGCCTACCCCTACAGA) and RNA667-REV (TATTGTCTTCCCAATCCTCCC) for the CRE and RNA 667 respectively. cDNA was precipitated in ethanol and amplified by PCR using primers HCV9181 (GGGCAGTAAGGACCAAGCTCAAA) and asHCV9359 for CRE and RNA667-FW (CTCGACTGTGCCTTCTAGTT) and RNA667-REV for RNA 667. Lysates and RNA templates (30 fmol) were subjected to RT-PCR in parallel. Samples were resolved in a 2% agarose gel.

### Luciferase measurement in HCV CRE-containing RNA constructs

Huh-7 cells were seeded in 24-well plates and allowed to grow until reach 80% confluence. Cells were transfected with siRNAs against the mRNAs of selected proteins as indicated above. After 48 h cells were re-transfected with RNA constructs containing *Firefly* or *Renilla* luciferase reporter genes and translation efficiency measured after 4 h using Dual Luciferase Reported Assay System (Promega). Translation efficiency was determined by the ratio FLuc/RLuc (*Firefly* luciferase /*Renilla* luciferase.

## Additional Information

**How to cite this article**: Ríos-Marco, P. *et al.* The *cis*-acting replication element of the Hepatitis C virus genome recruits host factors that influence viral replication and translation. *Sci. Rep.* **6**, 25729; doi: 10.1038/srep25729 (2016).

## Supplementary Material

Supplementary Information

Supplementary Dataset 1

## Figures and Tables

**Table 1:** LC-MS/MS identification of proteins pulled-down with HCV CRE.

Protein name	Name	Accession	M	P	U.P	Cov.	Ratio	HCV gen.	Ref.
40S ribosomal protein SA (Fragment)	RPSA	C9J9K3	30	3	3	15.00	1.00 : 0.35	IRES	61
Actin. cytoplasmic 1	ACTB	P60709	42	21	4	40.18	1.00 : 0.34		
Activated RNA polymerase II transcriptional coactivator p15	SUB1	P53999	14	10	10	43.31	1.00 : 0.02	3′UTR	26
Alpha-enolase	ENO1	P06733	47	6	4	16.53	1.00 : 0.06		
AT-rich interactive domain-containing protein 3A	ARID3A	Q99856	63	9	8	14.45	1.00 : 0.20		
Bifunctional polynucleotide phosphatase/kinase	PNKP	Q96T60	57	22	19	38.10	1.00 : 0.01		
Cellular nucleic acid-binding protein (isoform 2)	CNBP	P62633-2	19	7	6	35.68	1.00 : 0.04		
DAZ-associated protein 1	DAZPA1	Q96EP5	43	6	5	18.42	1.00 : 0.05		
DNA ligase 3	LIG3	P49916	113	17	13	16.60	1.00 : 0.04		
DNA polymerase beta	POLB	P06746	38	8	7	24.25	1.00 : 0.01	3′UTR	26
DNA repair protein XRCC1	XRCC1	P18887	69	20	17	24.76	1.00 : 0.02		
DNA-(apurinic or apyrimidinic site) lyase	APEX1	P27695	36	5	4	19.81	1.00 : 0.00		
Embryonic stem cell-specific 5-hydroxymethylcytosine-binding protein	HMCES	Q96FZ2	41	9	9	27.68	1.00 : 0.03		
Eukaryotic translation initiation factor 3 subunit I	EIF3I	Q13347	37	5	4	17.00	1.00 : 0.21		
Exosome complex component RRP41	EXOSC4	Q9NPD3	26	5	4	20.91	1.00 : 0.04		
Far upstream element-binding protein 3	FUBP3	Q96I24	62	7	6	14.90	1.00 : 0.28		
Fructose-bisphosphate aldolase A	ALDOA	P04075	39	7	6	18.04	1.00 : 0.11		
Glyceraldehyde-3-phosphate dehydrogenase	GAPDH	P04406	36	9	8	23.78	1.00 : 0.05	3′UTR	26
Helicase-like transcription factor	HLTF	Q14527	114	27	25	22.94	1.00 : 0.04		
Heterogeneous nuclear ribonucleoprotein A/B	HNRNPAB	D6RD18	30	5	4	21.27	1.00 : 0.02		
Heterogeneous nuclear ribonucleoprotein A0	HNRNPA0	Q13151	31	9	8	26.88	1.00 : 0.03	IRES	61
Heterogeneous nuclear ribonucleoprotein A1	HNRNPA1	F8W6I7	33	30	16	53.58	1.00 : 0.03	3′UTR IRES	26 48 61
Heterogeneous nuclear ribonucleoprotein A3	HNRNPA3	P51991	40	19	17	34.92	1.00 : 0.02		
Heterogeneous nuclear ribonucleoprotein D-like (isoform 3)	HNRNPDL	O14979-3	28	5	4	22.75	1.00 : 0.06	3′UTR IRES	26 61
Heterogeneous nuclear ribonucleoprotein M (isoform 2)	HNRNPM	P52272-2	74	65	14	50.28	1.00 : 0.11		
Heterogeneous nuclear ribonucleoprotein U	HNRNPU	Q00839	91	24	21	21.72	1.00 : 0.08	3′UTR	26
Heterogeneous nuclear ribonucleoproteins A2/B1	HNRNPA2B1	P22626	37	20	17	39.94	1.00 : 0.04		
High mobility group protein B1	HMGB1	P09429	25	9	5	36.51	1.00 : 0.01	IRES	61
High mobility group protein B2	HMGB2	P26583	24	14	11	41.63	1.00 : 0.02		
Isocitrate dehydrogenase [NADP] cytoplasmic	IDH1	O75874	47	19	16	39.37	1.00 : 0.27		
Lamina-associated polypeptide 2. isoform alpha	TMPO	P42166	75	9	6	16.85	1.00 : 0.08		
L-lactate dehydrogenase A chain	LDHA	P00338	37	11	9	31.70	1.00 : 0.18		
Poly [ADP-ribose] polymerase 1	PARP1	P09874	113	53	51	36.61	1.00 : 0.02	3′UTR	26
Probable ATP-dependent RNA helicase DDX5	DDX5	P17844	69	8	6	15.06	1.00 : 0.30	3′UTR	26
Proteasome subunit alpha type-1	PSMA1	P25786	30	4	3	18.72	1.00 : 0.14		
Putative RNA-binding protein 3	RBM3	P98179	17	7	7	36.94	1.00 : 0.04	IRES	61
Ras GTPase-activating protein-binding protein 1	G3BP1	Q13283	52	13	12	26.10	1.00 : 0.31		
Ras GTPase-activating protein-binding protein 2 (isoform B)	G3BP2	Q9UN86-2	51	10	9	17.87	1.00 : 0.11		
Replication protein A 14 kDa subunit	RPA3	P35244	14	3	3	31.33	1.00 : 0.02		
Replication protein A 32 kDa subunit (isoform 3)	RPA2	P15927-3	39	6	5	16.11	1.00 : 0.00		
Replication protein A 70 kDa DNA-binding subunit	RPA1	P27694	68	24	19	31.69	1.00 : 0.02		
RNA-binding motif. single-stranded-interacting protein 1	RBMS1	E7ETU5	46	5	5	16.80	1.00 : 0.05		
RNA-binding protein 7	RBM7	Q9Y580	31	5	4	17.01	1.00 : 0.02		
RNA-binding protein Musashi homolog 1	MSI1	O43347	39	7	5	18.32	1.00 : 0.04		
RNA-binding protein Musashi homolog 2 (isoform 2)	MSI2	Q96DH6-2	28	11	8	34.66	1.00 : 0.04	IRES	61
Single-stranded DNA-binding protein (Fragment)	SSBP1	E7EUY5	16	3	3	27.74	1.00 : 0.14	IRES	61
Splicing factor. proline- and glutamine-rich	SFPQ	P23246	76	14	13	19.48	1.00 : 0.29	3′UTR	26
T-complex protein 1 subunit theta	CCT8	P50990	60	13	12	25.59	1.00 : 0.25		
Thymidylate kinase	DTYMK	P23919	24	7	4	30.50	1.00 : 0.02		
Transcriptional activator protein Pur-beta	PURB	Q96QR8	33	5	4	16.26	1.00 : 0.04		
X-ray repair cross-complementing protein 5	XRCC5	P13010	83	24	22	26.74	1.00 : 0.15		
X-ray repair cross-complementing protein 6	XRCC6	P12956	70	29	28	35.22	1.00 : 0.15	3′UTR IRES	26 61
YTH domain family protein 1	YTHDF1	Q9BYJ9	61	14	5	25.51	1.00 : 0.09		
YTH domain family protein 2	YTHDF2	Q9Y5A9	62	14	10	25.82	1.00 : 0.11		
YTH domain family protein 3	YTHDF3	Q7Z739	64	18	7	32.88	1.00 : 0.10		

Name, gene name; Accession, Uniprot accession number; M, molecular mass (kDa); P, number of peptides identified in the positive samples; U.P, number of unique peptides identified in the positive samples; Cov., coverage of full-length protein in positive samples by tryptic peptides (%); Ratio, average of peak area ratios from peptides in positive samples; compared to control samples; HCV gen., other reported HCV genome domains which associate with the protein; Ref., references for the named interactions.

**Table 2:** Functional annotation clustering of CRE-interacting candidates.

ANNOTATION CLUSTER	SCORE	ANNOTATION CATEGORY	TERMS	N	P.VALUE	BENJAMINI
Cluster 1	15.9	INTERPRO	RNA recognition motif, RNP-1	16	1.1E-16	1.4E-14
	15.9	INTERPRO	Nucleotide-binding, alpha-beta plait	16	1.3E-16	6.8E-15
	15.9	SMART	RRM	16	1.3E-16	2.2E-15
Cluster 2	8.87	GOTERM_BP_FAT	DNA repair	14	2.7E-11	1.2E-8
	8.87	GOTERM_BP_FAT	response to DNA damage stimulus	14	8.0E-10	1.2E-7
	8.87	GOTERM_BP_FAT	cellular response to stress	14	1.2E-7	1.0E-5
Cluster 3	8.81	GOTERM_CC_FAT	intracellular organelle lumen	24	1.0E-9	3.8E-8
	8.81	GOTERM_CC_FAT	organelle lumen	24	1.6E-9	4.6E-8
	8.81	GOTERM_CC_FAT	membrane-enclosed lumen	24	2.4E-9	5.4E-8
Cluster 4	4.91	GOTERM_CC_FAT	DNA replication factor A complex	4	7.0E-7	8.9E-6
	4.91	GOTERM_CC_FAT	replisome	4	1.3E-5	1.3E-4
	4.91	GOTERM_CC_FAT	nuclear replisome	4	1.3E-5	1.3E-4
	4.91	GOTERM_CC_FAT	nuclear replication fork	4	1.6E-5	1.3E-4
	4.91	GOTERM_CC_FAT	replication fork	4	1.6E-4	1.0E-3
Cluster 5	3.71	GOTERM_BP_FAT	RNA splicing	8	8.8E-5	3.6E-3
	3.71	GOTERM_BP_FAT	mRNA processing	8	1.9E-4	7.0E-3
	3.71	GOTERM_BP_FAT	mRNA metabolic process	8	4.4E-4	1.3E-2
Cluster 6	3.57	GOTERM_BP_FAT	RNA splicing, via transesterification reactions	6	2.7E-4	9.2E-3
	3.57	GOTERM_BP_FAT	RNA splicing, via transesterification reactions with bulged adenosine as nucleophile	6	2.7E-4	9.2E-3
	3.57	GOTERM_BP_FAT	nuclear mRNA splicing, via spliceosome	6	2.7E-4	9.2E-3
Cluster 7	3.42	KEGG_PATHWAY	Mismatch repair	4	2.0E-4	3.0E-3
	3.42	KEGG_PATHWAY	Homologous recombination	4	3.6E-4	3.7E-3
	3.42	KEGG_PATHWAY	DNA replication	4	7.6E-4	5.9E-3
Cluster 8	3.3	GOTERM_BP_FAT	nucleotide-excision repair, DNA damage removal	4	7.4E-5	3.3E-3
	3.3	GOTERM_BP_FAT	nucleotide-excision repair	4	1.2E-3	2.8E-2
	3.3	GOTERM_BP_FAT	DNA catabolic process	4	1.5E-3	3.3E-2

SCORE, enrichment score of the annotation cluster; N, number of genes involved in the term.

## References

[b1] Choo Q. L. *et al.* Isolation of a cDNA clone derived from a blood-borne non-A, non-B viral hepatitis genome. Science 244, 359–362 (1989). 10.1126/science.2523562 2523562

[b2] Takamizawa A. *et al.* Structure and organization of the hepatitis C virus genome isolated from human carriers. J. Virol. 65, 1105–1113 (1991). 1847440 10.1128/jvi.65.3.1105-1113.1991 PMC239876

[b3] Tsukiyama-Kohara K., Iizuka N., Kohara M. & Nomoto A. Internal ribosome entry site within hepatitis C virus RNA. J. Virol 66, 1476–1483 (1992). 10.1128/jvi.66.3.1476-1483.1992 PMC240872 1310759

[b4] Wang C., Sarnow P. & Siddiqui A. Translation of human hepatitis C virus RNA in cultured cells is mediated by an internal ribosome-binding mechanism. J Virol. 67, 3338–3344 (1993). 10.1128/jvi.67.6.3338-3344.1993 PMC237677 8388503

[b5] Kolykhalov A. A., Mihalik K., Feinstone S. M. & Rice C. M. Hepatitis C virus-encoded enzymatic activities and conserved RNA elements in the 3′ nontranslated region are essential for virus replication *in vivo*. J. Virol. 74, 2046–2051 (2000). 10644379 10.1128/jvi.74.4.2046-2051.2000 PMC111684

[b6] Friebe P. & Bartenschlager R. Genetic analysis of sequences in the 3′ nontranslated region of hepatitis C virus that are important for RNA replication. J. Virol. 76, 5326–5338 (2002). 10.1128/JVI.76.11.5326-5338.2002 PMC137049 11991961

[b7] Yi M. & Lemon S. M. 3′ nontranslated RNA signals required for replication of hepatitis C virus RNA. J.Virol. 77, 3557–3568 (2003). 10.1128/JVI.77.6.3557-3568.2003 PMC149512 12610131

[b8] Blight K. J. & Rice C. M. Secondary structure determination of the conserved 98-base sequence at the 3′ terminus of hepatitis C virus genome RNA. J. Virol. 71, 7345–7352 (1997). 10.1128/jvi.71.10.7345-7352.1997 PMC192079 9311812

[b9] Oh J. W., Sheu G. T. & Lai M. M. Template requirement and initiation site selection by hepatitis C virus polymerase on a minimal viral RNA template. J. Biol. Chem. 275, 17710–17717 (2000). 10.1074/jbc.M908781199 10749880

[b10] Kanai A., Tanabe K. & Kohara M. Poly(U) binding activity of hepatitis C virus NS3 protein, a putative RNA helicase. FEBS Lett. 376, 221–224 (1995). 10.1016/0014-5793(95)01283-x 7498546

[b11] Huang L. *et al.* Hepatitis C virus non-structural protein 5A (NS5A) is a RNA-binding protein. J. Biol. Chem. 280, 36417–36428 (2005). 10.1074/jbc.M508175200 16126720

[b12] Tingting P., Caiyun F., Zhigang Y., Pengyuan Y. & Zhenghong Y. Subproteomic analysis of the cellular proteins associated with the 3′ untranslated region of the hepatitis C virus genome in human liver cells. Biochem Biophys Res. Commun. 347, 683–691 (2006). 10.1016/j.bbrc.2006.06.144 16842740

[b13] Isken O. *et al.* Nuclear factors are involved in hepatitis C virus RNA replication. RNA 13, 1675–1692 (2007). 10.1261/rna.594207 PMC1986813 17684232

[b14] McCaffrey A. P. *et al.* Determinants of hepatitis C translational initiation *in vitro*, in cultured cells and mice. Mol. Ther. 5, 676–684 (2002). 10.1006/mthe.2002.0600 12027551

[b15] Bung C. *et al.* Influence of the hepatitis C virus 3′-untranslated region on IRES-dependent and cap-dependent translation initiation. FEBS Lett. 584, 837–842 (2010). 10.1016/j.febslet.2010.01.015 20079737

[b16] Tuplin A., Struthers M., Cook J., Bentley K. & Evans D. J. Inhibition of HCV translation by disrupting the structure and interactions of the viral CRE and 3′ X-tail. Nucleic Acids Res. 43, 2914–2926 (2015). 10.1093/nar/gkv142 PMC4357731 25712095

[b17] Lee H., Shin H., Wimmer E. & Paul A. V. cis-acting RNA signals in the NS5B C-terminal coding sequence of the hepatitis C virus genome. J. Virol. 78, 10865–10877 (2004). 10.1128/JVI.78.20.10865-10877.2004 PMC521798 15452207

[b18] Tuplin A., Evans D. J. & Simmonds P. Detailed mapping of RNA secondary structures in core and NS5B-encoding region sequences of hepatitis C virus by RNase cleavage and novel bioinformatic prediction methods. J. Gen. Virol. 85, 3037–3047 (2004). 10.1099/vir.0.80141-0 15448367

[b19] You S., Stump D. D., Branch A. D. & Rice C. M. A cis-acting replication element in the sequence encoding the NS5B RNA-dependent RNA polymerase is required for hepatitis C virus RNA replication. J. Virol. 78, 1352–1366 (2004). 10.1128/JVI.78.3.1352-1366.2004 PMC321395 14722290

[b20] Friebe P., Boudet J., Simorre J. P. & Bartenschlager R. Kissing-loop interaction in the 3′ end of the hepatitis C virus genome essential for RNA replication. J. Virol. 79, 380–392 (2005). 10.1128/JVI.79.1.380-392.2005 PMC538730 15596831

[b21] Diviney S. *et al.* A hepatitis C virus cis-acting replication element forms a long-range RNA-RNA interaction with upstream RNA sequences in NS5B. J. Virol. 82, 9008–9022 (2008). 10.1128/JVI.02326-07 PMC2546899 18614633

[b22] Romero-Lopez C. & Berzal-Herranz A. The functional RNA domain 5BSL3.2 within the NS5B coding sequence influences hepatitis C virus IRES-mediated translation. Cell. Mol. Life Sci. 69, 103–113 (2012). 10.1007/s00018-011-0729-z PMC11115049 21598019

[b23] Lu L. *et al.* Mutations conferring resistance to a potent hepatitis C virus serine protease inhibitor *in vitro*. Antimicrob. Agents Chemother. 48, 2260–2266 (2004). 10.1128/AAC.48.6.2260-2266.2004 PMC415624 15155230

[b24] Oakland T. E., Haselton K. J. & Randall G. EWSR1 binds the hepatitis C virus cis-acting replication element and is required for efficient viral replication. J. Virol. 87, 6625–6634 (2013). 10.1128/JVI.01006-12 PMC3676111 23552423

[b25] Chien H. L., Liao C. L. & Lin Y. L. FUSE binding protein 1 interacts with untranslated regions of Japanese encephalitis virus RNA and negatively regulates viral replication. J. Virol. 85, 4698–4706 (2011). 10.1128/JVI.01950-10 PMC3126168 21367899

[b26] Harris D., Zhang Z., Chaubey B. & Pandey V. N. Identification of cellular factors associated with the 3′-nontranslated region of the hepatitis C virus genome. Mol. Cell Proteomics 5, 1006–1018 (2006). 10.1074/mcp.M500429-MCP200 16500930

[b27] Huang da W., Sherman B. T. & Lempicki R. A. Systematic and integrative analysis of large gene lists using DAVID bioinformatics resources. Nature Protocols 4, 44–57 (2009). 10.1038/nprot.2008.211 19131956

[b28] Agostini F. *et al.* catRAPID omics: a web server for large-scale prediction of protein-RNA interactions. Bioinformatics 29, 2928–2930 (2013). 10.1093/bioinformatics/btt495 PMC3810848 23975767

[b29] Jagan I., Fatehullah A., Deevi R. K., Bingham V. & Campbell F. C. Rescue of glandular dysmorphogenesis in PTEN-deficient colorectal cancer epithelium by PPARgamma-targeted therapy. Oncogene 32, 1305–1315 (2013). 10.1038/onc.2012.140 PMC3446865 22543585

[b30] Le P. N., Maranon D. G., Altina N. H., Battaglia C. L. & Bailey S. M. TERRA, hnRNP A1, and DNA-PKcs Interactions at Human Telomeres. Frontiers in Oncology 3, 91 (2013). 10.3389/fonc.2013.00091 PMC3628365 23616949

[b31] Raaijmakers J. A., Tanenbaum M. E., Maia A. F. & Medema R. H. RAMA1 is a novel kinetochore protein involved in kinetochore-microtubule attachment. J. Cell Sci. 122, 2436–2445 (2009). 10.1242/jcs.051912 19549680

[b32] You S. & Rice C. M. 3′ RNA elements in hepatitis C virus replication: kissing partners and long poly(U). J Virol 82, 184–195 (2008). 10.1128/JVI.01796-07 PMC2224383 17942554

[b33] Romero-Lopez C. & Berzal-Herranz A. A long-range RNA-RNA interaction between the 5′ and 3′ ends of the HCV genome. RNA 15, 1740–1752 (2009). 10.1261/rna.1680809 PMC2743058 19605533

[b34] Maris C., Dominguez C. & Allain F. H. The RNA recognition motif, a plastic RNA-binding platform to regulate post-transcriptional gene expression. FEBS J. 272, 2118–2131 (2005). 10.1111/j.1742-4658.2005.04653.x 15853797

[b35] Kumar A., Ray U. & Das S. Human La protein interaction with GCAC near the initiator AUG enhances hepatitis C Virus RNA replication by promoting linkage between 5′ and 3′ untranslated regions. J. Virol. 87, 6713–6726, 10.1128/JVI.00525-13 (2013). PMC3676093 23552417

[b36] Bell A. J. Jr., Chauhan S., Woodson S. A. & Kallenbach N. R. Interactions of recombinant HMGB proteins with branched RNA substrates. Biochem. Biophys. Res. Commun. 377, 262–267 (2008). 10.1016/j.bbrc.2008.09.131 PMC10587908 18845125

[b37] Hamilton B. J., Burns C. M., Nichols R. C. & Rigby W. F. Modulation of AUUUA response element binding by heterogeneous nuclear ribonucleoprotein A1 in human T lymphocytes. The roles of cytoplasmic location, transcription, and phosphorylation. J. Biol. Chem. 272, 28732–28741 (1997). 10.1074/jbc.272.45.28732 9353343

[b38] Morton S. *et al.* Phosphorylation of the ARE-binding protein DAZAP1 by ERK2 induces its dissociation from DAZ. Biochem. J. 399, 265–273 (2006). 10.1042/BJ20060681 PMC1609909 16848763

[b39] Rousseau S. *et al.* Inhibition of SAPK2a/p38 prevents hnRNP A0 phosphorylation by MAPKAP-K2 and its interaction with cytokine mRNAs. EMBO 21, 6505–6514 (2002). 10.1093/emboj/cdf639 PMC136943 12456657

[b40] Cok S. J., Acton S. J., Sexton A. E. & Morrison A. R. Identification of RNA-binding proteins in RAW 264.7 cells that recognize a lipopolysaccharide-responsive element in the 3-untranslated region of the murine cyclooxygenase-2 mRNA. J. Biol. Chem. 279, 8196–8205 (2004). 10.1074/jbc.M308475200 14662769

[b41] Rocak S. & Linder P. DEAD-box proteins: the driving forces behind RNA metabolism. Nature Rev. 5, 232–241 (2004). 10.1038/nrm1335 14991003

[b42] Sen N. D., Zhou F., Ingolia N. T. & Hinnebusch A. G. Genome-wide analysis of translational efficiency reveals distinct but overlapping functions of yeast DEAD-box RNA helicases Ded1 and eIF4A. Genome Res. 25, 1196–1205 (2015). 10.1101/gr.191601.115 PMC4510003 26122911

[b43] Gibson B. A. & Kraus W. L. New insights into the molecular and cellular functions of poly(ADP-ribose) and PARPs. Nature Rev. 13, 411–424 (2012). 10.1038/nrm3376 22713970

[b44] Jung J. H. *et al.* Hepatitis C virus infection is blocked by HMGB1 released from virus-infected cells. J. Virol. 85, 9359–9368 (2011). 10.1128/JVI.00682-11 PMC3165778 21752923

[b45] Ward A. M. *et al.* Quantitative mass spectrometry of DENV-2 RNA-interacting proteins reveals that the DEAD-box RNA helicase DDX6 binds the DB1 and DB2 3′ UTR structures. RNA Biol. 8, 1173–1186 (2011). 10.4161/rna.8.6.17836 PMC3256426 21957497

[b46] Garaigorta U., Heim M. H., Boyd B., Wieland S. & Chisari F. V. Hepatitis C virus (HCV) induces formation of stress granules whose proteins regulate HCV RNA replication and virus assembly and egress. J. Virol. 86, 11043–11056 (2012). 10.1128/JVI.07101-11 PMC3457181 22855484

[b47] Yi Z. *et al.* Hepatitis C virus co-opts Ras-GTPase-activating protein-binding protein 1 for its genome replication. J. Virol. 85, 6996–7004 (2011). 10.1128/JVI.00013-11 PMC3126560 21561913

[b48] Kim C. S., Seol S. K., Song O. K., Park J. H. & Jang S. K. An RNA-binding protein, hnRNP A1, and a scaffold protein, septin 6, facilitate hepatitis C virus replication. J. Virol. 81, 3852–3865 (2007). 10.1128/JVI.01311-06 PMC1866118 17229681

[b49] Marton S., Romero-Lopez C. & Berzal-Herranz A. RNA aptamer-mediated interference of HCV replication by targeting the CRE-5BSL3.2 domain. J. Viral Hepat. 20, 103–112 (2013). 10.1111/j.1365-2893.2012.01629.x 23301545

[b50] Guo R. *et al.* HnRNP A1/A2 and SF2/ASF regulate alternative splicing of interferon regulatory factor-3 and affect immunomodulatory functions in human non-small cell lung cancer cells. PloS one 8, e62729 (2013). 10.1371/journal.pone.0062729 PMC3639176 23658645

[b51] Romero-Lopez C., Diaz-Gonzalez R. & Berzal-Herranz A. Inhibition of hepatitis C virus internal ribosome entry site-mediated translation by an RNA targeting the conserved IIIf domain. Cell. Mol. Life Sci. 64, 2994–3006 (2007). 10.1007/s00018-007-7345-y PMC11136273 17938858

[b52] Romero-Lopez C., Barroso-Deljesus A., Garcia-Sacristan A., Briones C. & Berzal-Herranz A. The folding of the hepatitis C virus internal ribosome entry site depends on the 3′-end of the viral genome. Nucleic Acids Res. 40, 11697–11713 (2012). 10.1093/nar/gks927 PMC3526292 23066110

[b53] Larrea E. *et al.* Altered expression and activation of signal transducers and activators of transcription (STATs) in hepatitis C virus infection: *in vivo* and *in vitro* studies. Gut 55, 1188–1196 (2006). 10.1136/gut.2005.070060 PMC1856287 16120756

[b54] Romero-Lopez C., Diaz-Gonzalez R., Barroso-Deljesus A. & Berzal-Herranz A. Inhibition of HCV replication and IRES-dependent translation by an RNA molecule. J. Gen. Virol. 90, 1659–1669 (2009). 10.1099/vir.0.008821-0 19264618

[b55] Lopez de Quinto S. & Martinez-Salas E. Interaction of the eIF4G initiation factor with the aphthovirus IRES is essential for internal translation initiation *in vivo*. RNA 6, 1380–1392 (2000). 10.1017/s1355838200000753 PMC1370009 11073214

[b56] Shevchenko A., Wilm M., Vorm O. & Mann M. Mass spectrometric sequencing of proteins silver-stained polyacrylamide gels. Anal. Chem. 68, 850–858 (1996). 10.1021/ac950914h 8779443

[b57] Moreno M. L. *et al.* Disulfide stress: a novel type of oxidative stress in acute pancreatitis. Free Radical Biol. Med. 70, 265–277 (2014). 10.1016/j.freeradbiomed.2014.01.009 24456905

[b58] Perez M. *et al.* Mutual regulation between SIAH2 and DYRK2 controls hypoxic and genotoxic signaling pathways. J.Mol. Cell Biol. 4, 316–330 (2012). 10.1093/jmcb/mjs047 22878263

[b59] Mi H., Muruganujan A., Casagrande J. T. & Thomas P. D. Large-scale gene function analysis with the PANTHER classification system. Nature Protocols 8, 1551–1566 (2013). 10.1038/nprot.2013.092 PMC6519453 23868073

[b60] Niranjanakumari S., Lasda E., Brazas R. & García-Blanco M. A. Reversible cross-linking combined with immunoprecipitation to study RNA-protein interactions *in vivo*. Methods 26, 182–190 (2002). 10.1016/S1046-2023(02)00021-X 12054895

[b61] Lu H., Li W., Noble W. S., Payan D. & Anderson D. C. Riboproteomics of the hepatitis C virus internal ribosomal entry site. J.Proteome Res. 3, 949–957 (2004). 10.1021/pr0499592 15473682

