# Supplementary material for: The cis-acting replication element of the Hepatitis C virus genome recruits host factors that influence viral replication and translation
Source: Sci Rep. 2016 May 11;6:25729. doi: 10.1038/srep25729 (PMC4863150; doi:10.1038/srep25729)
Supplement: Supplementary Information [file srep25729-s1.doc]

**The *cis*-acting replication element of the Hepatitis C virus genome recruits host factors that influence viral replication and translation**

Pablo Ríos-Marco, Cristina Romero-López, Alfredo Berzal-Herranz^*^

Instituto de Parasitología y Biomedicina López-Neyra, (IPBLN-CSIC). PTS Granada, Avda. del Conocimiento s/n, Armilla, 18016 Granada, Spain.

^*^To whom correspondence should be addressed.

Instituto de Parasitología y Biomedicina López-Neyra, (IPBLN-CSIC).

PTS Granada, Avda. del Conocimiento s/n, Armilla, 18016 Granada, Spain

Tel: +34 958 181 648; Fax: +34 958 181 632

email: aberzalh@ipb.csic.es

**Supplementary Information**

**Tandem mass liquid chromatography protocol**

The precipitated samples were resuspended in sample buffer and loaded onto a polyacrylamide gel. The run was stopped as soon as the front entered the resolving gel. The protein bands were visualized by Coomassie staining, excised, and the gel pieces destained in acetonitrile:water (ACN:H_2_O, 1:1). The gel pieces were shrunk, dried in a SpeedVac dessicator, and re-swollen in 50 mM ammonium bicarbonate pH 8.8 with 60 ng/µl trypsin*.* The tubes were kept in ice for 2 h and incubated at 37°C for 12 h. Digestion was stopped by the addition of 1% trifluor acetic acid. Whole supernatants were dried down and then desalted onto OMIX C18 pipette tips until LC-MS/MS.

The desalted protein digest was dried, resuspended in 0.1% formic acid and analyzed by reverse phase LC-MS/MS in an Easy-nLC II system coupled to an ion trap LTQ-Orbitrap-Velos-Pro mass spectrometer (Thermo Fisher Scientific)*.* The peptides were concentrated (on-line) by reverse phase chromatography using an Acclaim PepMap C180 1 mm × 20 mm precolumn (5 µm, 100 A) (Thermo Fisher Scientific), and then separated using an Acclaim PepMap C18 0.075 mm x 150 mm column (3 µm particle size, 100 A pore size) (Thermo Fisher Scientific). The digested peptides were separated over 90 min in a 5-40% gradient of B solvent in A solvent (A Solvent: 0.1% formic acid in water, B solvent: 0.1% formic acid, 80% acetonitrile in water). Electrospray ionization was performed using a nanobore stainless steel emitter (internal diameter 30 μm) (Thermo Fisher Scientific) interface. The Orbitrap resolution was set at 30,000. Peptides were detected in survey scans from 400 to 1600 amu (1 μ scan), followed by 20 data dependent MS/MS scans (Top 20), using an isolation width of 2 u (in mass-to-charge ratio units), a normalized collision energy of 35%, and with dynamic exclusion applied for 30 s periods. Peptide identification from the raw data was performed using the SEQUEST algorithm (Proteome Discoverer 1.3, Thermo Fisher Scientific). The Uniprot-Homo database was searched using the following constraints: tryptic cleavage after Arg and Lys, up to two missed cleavage sites, and tolerances of 10 ppm for precursor ions and 0.8 Da for MS/MS fragment ions. All searches were performed allowing optional Met oxidation and Cys carbamidomethylation. To avoid false positives, a search was made of a decoy database, using the integrated decoy technique (false discovery rate [FDR] threshold <0.01). Raw data of samples are available upon request. Only proteins with two or more unique peptides were further analyzed. The outputs for the eight technical replicates were compared two by two using PEAKS 6 software. The label free quantification was based on the relative intensities of extracted ion chromatograms for precursor ions of identified peptides in the compared data sets. The protein ratios were calculated as average of the signal intensities (peak area) ratio from correlated peptide features between samples.
